# Drivers of SARS-CoV-2 testing behaviour: a modelling study using nationwide testing data in England

**DOI:** 10.1038/s41467-023-37813-1

**Published:** 2023-04-14

**Authors:** Younjung Kim, Christl A. Donnelly, Pierre Nouvellet

**Affiliations:** 1 Department of Evolution, Behaviour, and Environment, School of Life Sciences, University of Sussex, Brighton, UK; 2 Department of Statistics, University of Oxford, Oxford, UK; 3 Pandemic Sciences Institute, University of Oxford, Oxford, UK; 4 MRC Centre for Global Infectious Disease Analysis and Abdul Latif Jameel Institute for Disease and Emergency Analytics, Imperial College London, London, UK

**Keywords:** Epidemiology, Population screening, Health services, Computational models

## Abstract

During the COVID-19 pandemic, national testing programmes were conducted worldwide on unprecedented scales. While testing behaviour is generally recognised as dynamic and complex, current literature demonstrating and quantifying such relationships is scarce, despite its importance for infectious disease surveillance and control. Here, we characterise the impacts of SARS-CoV-2 transmission, disease susceptibility/severity, risk perception, and public health measures on SARS-CoV-2 PCR testing behaviour in England over 20 months of the pandemic, by linking testing trends to underlying epidemic trends and contextual meta-data within a systematic conceptual framework. The best-fitting model describing SARS-CoV-2 PCR testing behaviour explained close to 80% of the total deviance in NHS test data. Testing behaviour showed complex associations with factors reflecting transmission level, disease susceptibility/severity (e.g. age, dominant variant, and vaccination), public health measures (e.g. testing strategies and lockdown), and associated changes in risk perception, varying throughout the pandemic and differing between infected and non-infected people.

## Abstract (web summary)

SARS-CoV-2 testing rates have varied during the pandemic but the drivers of changes in testing behaviour are unclear. Here, the authors link national testing data from England to indicators of epidemic trends to describe how testing varies according to level of virus transmission, disease susceptibility/severity, public health measures, and risk perception.

## Introduction

During the COVID-19 pandemic, national SARS-CoV-2 testing programmes were conducted on an unprecedented scale across the world^1^. They played a significant role in monitoring and controlling SARS-CoV-2 transmission and mitigating its impact on public health. In most countries, contrasting with most testing for other infectious diseases, the majority of SARS-CoV-2 tests were conducted on a voluntary basis, making the interpretation of testing data challenging^2–4^. This highlights the importance of understanding factors driving testing behaviour^5,6^ in SARS-CoV-2 surveillance and control.

Currently, most studies interested in SARS-CoV-2 testing behaviour attempted either to characterise the willingness to be tested based on cross-sectional survey data^7–9^ or explored factors associated with actual SARS-CoV-2 testing behaviour based on longitudinal participatory survey data^10,11^. While providing a wealth of information at the individual level, as the authors acknowledged, such studies often suffered from selection bias, as survey participation is likely correlated to actual testing behaviour. Additionally, none of the studies accounted for fast-changing epidemiological contexts, such as transmission level, variant emergence, and vaccine rollout among other public health measures, although they could potentially bias study results when associated with variables of interest. In fact, throughout the pandemic, according to SARS-CoV-2 surveillance studies, most observed variability in positivity rate has been attributed to increased viral transmission within certain demographic groups^12,13^, which might have also led to heterogeneities in testing behaviour over time. Potentially due to this issue, the studies based on longitudinal survey data covered only a limited period (i.e. around 5 and 2 months, respectively)^10,11^. This led to a static view of factors influencing testing behaviour, although it is likely to have changed dynamically throughout the pandemic.

**Fig. 1:**
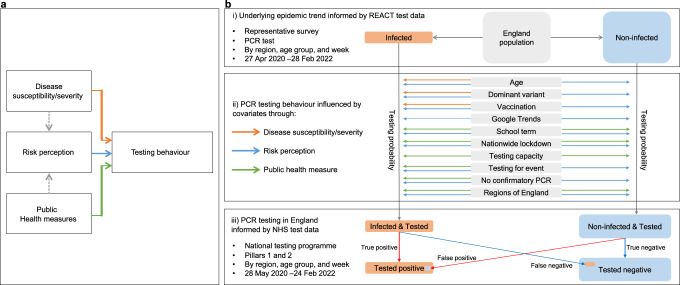
Conceptual framework for SARS-CoV-2 testing behaviour. **a** The relationships between mechanisms proposed to influence testing behaviour. The conceptual framework proposes that testing behaviour is influenced by the combined effect of (i) disease susceptibility/severity, (ii) risk perception, and (iii) public health measures (solid lines). Disease susceptibility/severity and public health measures are also proposed to influence risk perception (dotted lines), in addition to their direct impact on testing behaviour (solid lines). However, the indirect impact (i.e. dotted lines) could not be formally disentangled within our statistical framework. **b** Organisation of data and covariates under the conceptual framework. In models built on this framework, the population was divided into infected or non-infected people for each region of England, age group, and week, based on REACT test data (i). Then, infected or non-infected people were hypothesised to have taken a SARS-CoV-2 PCR test, depending on factors related to disease susceptibility/severity, risk perception, and public health measures (ii). Finally, the outcome of PCR test results was fitted to NHS test data by dynamic models (iii).

The above limitations suggest the need for a new methodological approach to better understand testing behaviour for COVID-19 or other diseases requiring public health responses at the regional, national, or global level. Importantly, this methodological approach would require a conceptual framework that systematically describes mechanisms underpinning testing behaviour and disentangles their interrelationships while accounting for the underlying epidemic trends. Therefore, we propose a conceptual framework for SARS-CoV-2 testing behaviour upon which this study is built (Fig. 1). In this framework, the level of viral transmission is the primary driver for SARS-CoV-2 testing with higher incidence leading to more testing^14^. Then, at a given epidemic point, individuals’ testing probability would be mainly determined by a combined effect of the following three mechanisms: (i) disease susceptibility/severity^5,7,15–20^, (ii) risk perception^5,7,15–17^, and (iii) public health measures^21^. Our conceptual framework proposes that those three mechanisms are interrelated with one another (Fig. 1a) and could act differentially on people’s testing behaviour depending on their infection status (Fig. 1b). First, disease susceptibility/severity from SARS-CoV-2 infection would influence testing propensity only among infected people, with experiencing more severe symptoms increasing testing propensity. Second, public health measures, such as testing capacity, testing strategies, and social distancing, would affect testing propensity equally or differentially among infected and non-infected people depending on their target population. Finally, based on various factors, including awareness of disease susceptibility/severity and public health measures in place, people would perceive the risk of SARS-CoV-2 infection and its consequences at different levels. This risk perception would equally impact the testing propensity of infected and non-infected people, considering that most people take a SARS-CoV-2 test without knowing their infection status at the time of testing.

Here, based on the above conceptual framework for SARS-CoV-2 testing, we aim to understand factors influencing age-specific, spatial, and temporal trends in SARS-CoV-2 testing in England between 28 May 2020 and 24 February 2022. More specifically, using publicly available population-level data for England disaggregated by regions and age groups over 92 weeks, our study seeks to link PCR test results from the National Health Service (NHS) Test and Trace^22^ (hereafter NHS test data) to testing behaviour within dynamic models. Our models characterise testing behaviour separately for infected (defined as SARS-CoV-2 shedding as determined by PCR) and non-infected (defined as no SARS-CoV-2 shedding as determined by PCR) people based on age- and region-specific level of SARS-CoV-2 circulation from a continuously running representative PCR survey (i.e. REal-time Assessment of Community Transmission (REACT) study, hereafter REACT test data)^23^. This modelling approach allows us to explicitly describe the process of seeking and receiving a test in relation to various contextual meta-data related to disease susceptibility/severity, risk perception, and public health measures, accounting for underlying epidemic trends.

## Results

### SARS-CoV-2 PCR testing and underlying epidemic trends

NHS test data for England gave weekly PCR test results in 9 regions and 4 age groups over 92 weeks, resulting in 3,312 data points. The weekly number of PCR tests in England varied greatly throughout the epidemic, ranging from 392,873 (18 to 24 Jun 2020) to 3,835,758 (16 to 22 Dec 2021). Of these, the proportion of positive tests also showed large variability, ranging from 0.01 (29 April to 5 May 2021) to 0.31 (30 Dec 2021 to 5 Jan 2022). On the other hand, REACT test data provided weekly PCR test results from random samples of the population of England generated by 18 REACT rounds over 74 weeks. The weekly number of PCR tests ranged from 10 to 105,698, with a median of 23,867 (with the very low numbers per week arising from individuals returning tests taken outside of the intended time interval). The weekly percentage of positive PCR test results ranged from 0 to 4.6%, with a median of 0.4%. The latter ranged from 0 to 15.3%, with a median of 0.3%, when stratified by age and region. Based on the mean prevalence of PCR swab positivity obtained by fitting a generalised additive model (GAM) to REACT test data (hereafter REACT GAM fit) (Fig. S1), we estimated the number of infected and non-infected people in each region and age group for the start date of each week defined in the NHS test data. In England, the estimated weekly number of infected people fluctuated significantly, ranging from 30,763 (17 Jul 2020) to 2,529,972 (13 Jan 2022). Reflecting a relatively low level of infection prevalence at the population level, the estimated weekly number of non-infected people remained relatively constant, fluctuating by less than 4.7% (from 54,020,166 to 56,519,375). The number of infected people estimated by the REACT GAM fit was larger than the observed number of positive tests in NHS test data, except for 33 out of 3,312 data points (1.0%). Most of those data points originated from the early period of Delta variant circulation between 17 June and 19 Aug 2021 (*n* = 23, 69.7%), notably in people aged 20 to 39 years (*n* = 16, 48.5%), and the 59-day interval between REACT rounds 13 and 14 that was much longer than the average between-round interval (median: 17 days, 1^st^–3^rd^ quantiles: 13 to 19 days).

### Associations with testing behaviour

**Fig. 2:**
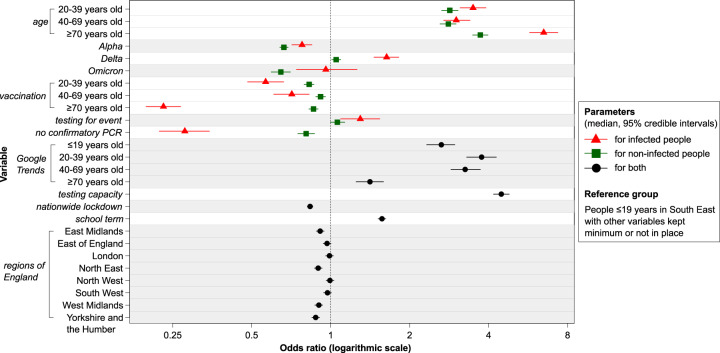
Odds ratios for taking a SARS-CoV-2 PCR test from the best-fitting model. The *x*-axis shows odds ratios on a logarithmic scale, and the *y*-axis shows variables included in the best-fitting model. Points and horizontal lines correspond to median and 95% credible intervals, respectively, estimated for the infected (red and triangle), non-infected (green and square), or both (black and circles). Infected (or non-infected) people aged ≤19 years (age group 1) in South East, with other variables kept minimum or not in place, represented the reference group for parameters estimated for infected (or non-infected) people. Parameters estimated for both infected and non-infected people had the same reference group with no differentiation by infection status. For testing capacity and Google Trends, odds ratios comparing the maximum and minimum values are shown. The models in this study were fitted to weekly NHS test data for England, which ranged from 392,873 (18 to 24 Jun 2020) to 3,835,758 (16 to 22 Dec 2021) tests over 92 weeks. See UK Health Security Agency^22^ for NHS test data.

Testing behaviour was associated with various factors related to the following three mechanisms allowed for by our conceptual framework: disease susceptibility/severity, risk perception, and public health measures (Figs. 1 and 2 and Tables S1 and S2).

First, the relationship between testing probability and variables linked to disease susceptibility/severity, i.e. age, dominance of the Alpha, Delta, and Omicron variants, and vaccination, typically followed the same direction for infected and non-infected people, but varied significantly in magnitude, likely reflecting the added impact of increased disease susceptibility/severity on testing behaviour among those infected (blue and orange arrows in Fig. 1).

In the best-fitting model, the probability of testing appeared to increase markedly with age in both infected and non-infected people (Fig. 2). The increase in testing probability was particularly substantial among infected people, being even more pronounced in people aged ≥70 years (OR: 6.49, 95%CrI: 5.73–7.34) compared with non-infected people (OR: 3.72, 95%CrI: 3.48–3.98), highlighting the impact of both disease susceptibility/severity and that of risk perception. The transmission of particular SARS-CoV-2 Variants of Concern^24^ was also associated with significant changes in testing behaviour. In particular, predominant circulation of the Delta variant was associated with increased testing, particularly among infected people (OR linked to ≥50% of circulating SARS-CoV-2 being Delta variant: 1.63, 95%CrI: 1.46–1.82). Contrastingly, predominant circulation of the Alpha and Omicron variant was associated with a decrease in testing, which was more pronounced among non-infected people (OR: 0.66, 95%Crl: 0.64–0.69 for the Alpha variant; OR: 0.65, 95%CrI: 0.59–0.70 for the Omicron variant).

Vaccination was associated with a decrease in testing behaviour among both infected and non-infected people and across age groups. The most substantial decrease in testing probability was among infected people, particularly in those aged ≥70 years (OR linked to ≥50% of the population receiving a second dose of vaccination: 0.23, 95%CrI: 0.20–0.27).

While the influence of variables described above was estimated differentially among infected and non-infected people by altering disease susceptibility/severity and risk perception, the following set of variables was allowed to affect equally or differentially infected and non-infected people through changes in risk perception (blue arrows in Fig. 1b) and/or public health measures (green arrows in Fig. 1b). COVID-19 social amplification measured by Google Trends as a proxy (search keyword: ‘covid’, normalised range: 0–100) appeared to play an essential role in driving testing behaviour, and the impact was least pronounced for people aged ≥70 years. The increase from the minimum (=14) to maximum (=100) Google Trends value was estimated to increase the odds of testing most in people aged 20 and 39 by 3.76 (95%CrI: 3.30–4.28) and least in those aged ≥70 by 1.41 (95%CrI: 1.25–1.59). The lateral flow device (LFD) testing requirement for attending large events appeared to increase PCR testing probability, with a significant increase among infected people (OR: 1.30, 95%CrI: 1.09–1.54). In contrast, PCR testing probability decreased when a confirmatory PCR test for people with a positive LFD test result was no longer required, especially among infected people (OR: 0.28, 95%CrI: 0.22–0.34). While both testing capacity and in-person school attendance were also associated with increased testing, the nationwide lockdown was associated with decreased testing. Finally, compared with South East, the probability of testing appeared to have been lower in other regions, including East Midlands, North East, Yorkshire and the Humber, and West Midlands.

### Contribution to explaining variability in NHS test data

**Fig. 3:**
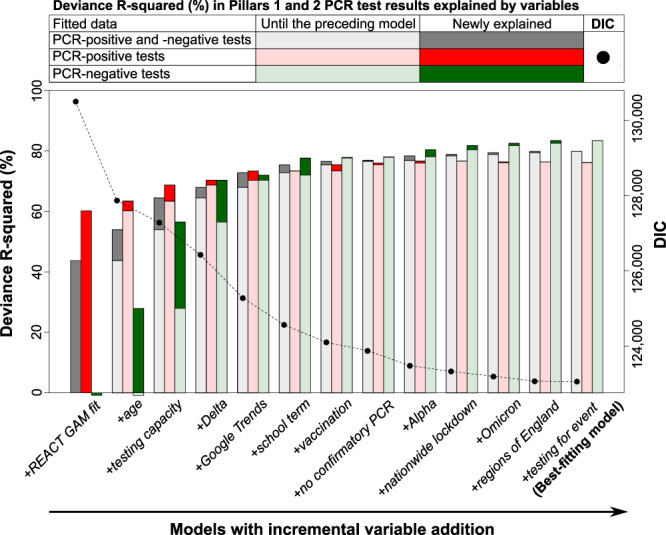
Deviance R-squared (%) explained during a forward stepwise selection procedure. Variables were added incrementally to the baseline model with only REACT GAM fit (most left) until the best-fitting model (most right). For each model, bars represent the total deviance R-squared explained by variables added in the preceding steps and the newly added variable (left *y*-axis). Different bar colours represent the deviance R-squared for PCR-positive tests (red), PCR-negative tests (green), or both (grey), with darker portions representing the deviance R-squared additionally explained by the newly added variable. Points represent the deviance information criterion (DIC) of each model (right *y*-axis).

**Fig. 4:**
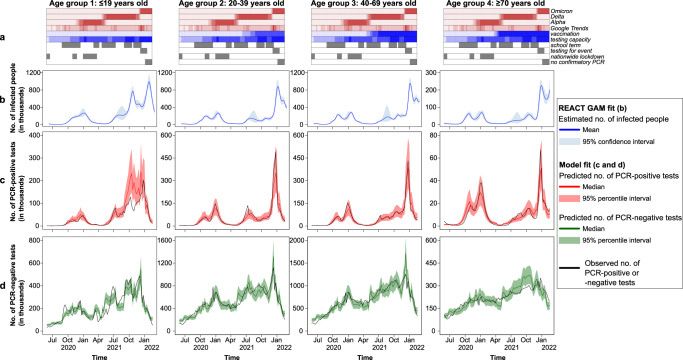
Predictive posterior check of the best-fitting model by age group and over time. Row **a** shows the temporal trend of variables included in the best-fitting model, and row **b** shows the temporal trend of the number of infected (mean, 95% confidence intervals) estimated by REACT GAM fit. Row **c** shows the predicted (red lines: median, reddish shades: 95% percentile intervals) and observed (black lines) numbers of Pillar 1 and Pillar 2 PCR-positive test results. Row **d** shows the predicted (green lines: median, greenish shades: 95% percentile intervals) and observed (black lines) numbers of Pillar 1 and Pillar 2 PCR-negative test results.

All variables included in the best-fitting model contributed to reducing the deviance information criterion (DIC) by more than 5 units (Fig. 3). The best-fitting model explained 79.8% of the total deviance in NHS test data, with marginally better performance for explaining the variability in the number of negative tests (83.4% explained) than positive tests (76.1% explained) (Fig. 3). For both positive and negative tests, the predicted numbers of PCR tests fitted reasonably well with the observed numbers over time by age group (Fig. 4 and S2) and region (Figs. S3 and S4).

Most of the variability in the number of positive tests was explained by the underlying epidemic trends (i.e. REACT GAM fit explained 60.2% of the deviance). To further explain variability in the number of positive tests, other important variables included testing capacity, age, and Google Trends, but their contributions to the overall deviance explained were relatively small (5.3%, 3.2%, and 3.0%, respectively). In contrast, explaining variability in the number of negative tests required accounting for more factors, notably age, testing capacity, and dominance of the Delta variant (28.7%, 28.6%, and 13.8%, respectively).

After accounting for variables in the best-fitting model, the probability of testing showed significant fluctuations throughout the epidemic (Fig. S5). Among infected people, the probability of testing gradually increased in all age groups, with intermittent drops. Notably, a sudden drop in testing probability was observed in people aged ≥70 when the population received a second dose of vaccination, whereas, in younger age groups, the largest drop was observed after the peak of the Omicron wave. Among those non-infected, the probability of testing also gradually increased over time until it decreased sharply after the peak of the Omicron wave in all age groups.

### Sensitivity analysis

Models with an alternative age classification in NHS test data and those with no age group weighting for the REACT GAM fit and vaccination did not substantially change the interpretation of the best-fitting model results (Fig. S6). More specifically, after including younger people in age group 4 (i.e. aged ≥60 years, instead of ≥70 years) (alternative model A), the effect of age and vaccination on testing behaviour decreased in age group 4, particularly among infected people, whereas the effect of Google Trends became more substantial in age group 4. Those associations remained significant with alternative model A. No age group weighting (alternative model B) resulted in no significant association with vaccination in age group 1, linked to having ≤17, instead of ≤19 years old in the age group. There were no other significant changes with alternative model B.

## Discussion

Our study results show complex dynamics of SARS-CoV-2 PCR testing behaviour, varying during 20 months of the pandemic in England and differing between infected and non-infected people, in association with factors hypothesised under our conceptual framework (Fig. 1).

One of the core hypotheses in our conceptual framework was that risk perception influenced the testing behaviour of both infected and non-infected people. Then, in addition to risk perception, factors related to disease susceptibility/severity, including age, SARS-CoV-2 virulence, and immune status, directly affected the testing behaviour of infected people by causing different levels of symptoms. Under this hypothesis, for a variable to affect testing behaviour by changing both disease susceptibility/severity and risk perception, the observed difference in the magnitude of associations between infected and non-infected people would reflect the relative contribution of disease susceptibility/severity and risk perception to testing behaviour, although, to quantify such an effect, one would need to know the precise form of the relationship of testing behaviour with those two mechanisms.

Indeed, considerable differences were observed for several variables between infected and non-infected people. The associations between testing behaviour and age in infected people, particularly among those aged ≥70 years, suggests that age-dependent susceptibility/severity to COVID-19^25,26^ served as the primary determinant of testing participation. Similar age group-specific patterns in non-infected people would indicate that different age groups formed different levels of risk perception based on their perceived risk of contracting COVID-19 and experiencing severe illness upon infection. In addition, people aged ≥70 years could have been more likely to take a test than those in younger age groups, if they had COVID-19-like symptoms, but not actually from SARS-CoV-2 infection, more frequently. Furthermore, people aged ≥70 years would have been prescribed a PCR test more often, for example, when admitted to hospitals or living in care homes. This would have further increased testing participation in this age group.

Our findings also suggest that people significantly changed their testing behaviour during the circulation of different dominant variants, potentially through changes in risk perception, in addition to heterogeneities in the variants’ virulence actually realised among those infected. For Delta variant circulation, a considerable increase in testing probability among infected people is likely to reflect the variant’s greater virulence^18,19,27^. Among non-infected people, the increase in testing probability may suggest that the Delta variant’s potential to cause severe disease increased testing participation by heightening risk perception. The same logic could explain the decrease in testing probability with Omicron variant circulation, given the variant’s association with generally milder and less specific symptoms^18,27^. While the association with Omicron variant circulation was significant among non-infected people, it was not among infected people. This was likely influenced by withdrawing a confirmatory PCR testing scheme in the middle of Omicron variant circulation, as it appeared to explain most of the decrease in testing participation in the best-fitting model.

For Alpha variant circulation, the decrease in testing probability was unexpected as the variant has been suggested to be more transmissible and cause more severe disease than pre-Alpha SARS-CoV-2 strains^20,28,29^. One possible explanation is the influence of epidemic trends on risk perception. The Alpha variant became dominant and peaked during the winter holiday season shortly after the first epidemic wave and the second nationwide lockdown. If risk perception was decreased by these factors, people could have become less willing to receive a test, offsetting the possible impact of the variant’s increased transmissibility and virulence. Importantly, this suggests that testing behaviour would not always follow the dynamics of viral circulation and virulence in the presence of other external factors.

With COVID-19 vaccination, the decrease in testing behaviour in both infected and non-infected people suggests that risk perception toward COVID-19 was reduced in the public with the widespread vaccine rollout. A greater decrease among infected people further indicates that vaccine effectiveness against symptomatic disease could have influenced the testing behaviour of those vaccinated upon infection^30–32^. Indeed, among infected people, the largest decrease was observed in those aged ≥70, who are most likely to experience symptomatic COVID-19 without vaccination^25,26^. This indicates that a significant fraction of this age group could have benefited from vaccination in avoiding apparent symptoms, thereby having considerably reduced test propensity. Our findings also suggest that efforts to increase testing participation should be maintained to control community transmission during widespread vaccine rollout because, while COVID-19 vaccination is proven to be effective for preventing severe disease^30–32^, its contribution to reducing SARS-CoV-2 transmissibility is becoming increasingly limited with the emergence of new variants^33,34^.

The increase in testing probability with Google Trends for the keyword ‘covid’ highlights the influence of COVID-19 social amplification on testing behaviour, potentially by affecting risk perception. The increase in testing probability was more pronounced in younger age groups, which might reflect different internet usage patterns between age groups^35^. For example, more frequent and interactive nature of digital media, rather than traditional media, could have affected risk perception and, thus, testing behaviour differently depending on internet usage patterns. However, there could also be other explanations not necessarily related to risk perception. First, compared with those aged ≥70 years, the testing behaviour in younger age groups could have been more responsive to COVID-19 social amplification, e.g. through increased use of online PCR test appointments, especially when the demand for PCR tests was high^36^. Secondly, if disease susceptibility/severity was a predominant factor determining testing behaviour among those aged ≥70 years, changes in the level of COVID-19 social amplification would have had limited influence on testing behaviour.

Our findings also suggest that testing behaviour was limited or driven by public health measures. Among them, testing capacity explained a relatively large fraction of the variability in NHS test data, particularly for the number of PCR-negative tests, confirming its importance for the design of the testing programmes. Moreover, its positive association with testing behaviour suggests that a shortage of available tests might have restricted testing participation, as witnessed at the beginning of the pandemic and some periods during the Omicron variant surge^37–39^. However, while increasing testing capacity would identify more infected people, it could diminish optimal use of limited testing resources if the increase in testing became disproportionately higher among non-infected people. This indicates that, if the primary objective of testing is to identify as many infected people as possible with a limited number of available tests, increasing testing capacity should be accompanied by strategies designed to increase the probability of testing infected people and therefore optimise the allocation of testing resources. Such strategies might include targeting high-risk groups, contact tracing, and effective risk communications.

Although public health measures introduced during particular periods showed an association with testing behaviour, they explained a relatively small proportion of the variability in NHS test data. First, during school terms, a confirmed COVID-19 case in a class would trigger testing students in the school, thereby increasing testing participation. In particular, the introduction of regular testing in schools in March 2021 likely facilitated this process^40^. Furthermore, during school terms, students could have re-evaluated the risk of contracting COVID-19 and therefore changed their testing behaviour. Similarly, requiring an LFD test for attending large events was also shown effective for increasing PCR testing probability, especially among infected people, likely from requiring a confirmatory PCR test on those who tested positive with an LFD test. This, in turn, explains the decrease in testing participation after withdrawing the confirmatory PCR test policy, particularly among infected people. Nationwide lockdowns were also negatively associated with testing participation, possibly reflecting a reduced need for testing from having fewer contacts and a reduced level of risk perception following a rapidly shirking epidemic trend during lockdown periods.

Finally, the probability of testing varied among regions. Interestingly, regions with a significantly lower testing probability—East Midlands, West Midlands, Yorkshire and the Humber, and North East— also have the lowest average household income in England; while the reference region, i.e. South East, has the second highest average household income, close to that of London^41^. This might suggest that socio-economic deprivation hindered testing participation to some extent during the pandemic. Other factors could have also contributed to regional differences in testing behaviour. In fact, despite having a relatively low average household income, North West showed a similar testing probability as the reference region. The relatively high testing probability in North West may have been affected by the voluntary mass LFD testing pilot conducted in Liverpool, one of the largest cities in the region, for people with or without COVID-19 symptoms^42^. Between 6 November 2020 and 30 April 2021, 57% of Liverpool residents took an LFD test, which led to an increase in PCR testing to confirm a positive LFD test result^43^. The awareness of this pilot may have also influenced risk perception and, therefore, PCR testing in the region. In addition, population size could have also impacted testing behaviour if accessibility to testing centres or perceived risk of contracting COVID-19 was associated with population size; South East and North West are ranked 1^st^ and 3^rd^ by population size, respectively^44^, whereas regions with a significantly lower testing probability have relatively small populations.

Several factors must be considered when interpreting the findings of this study. First, the key mechanisms proposed by our modelling framework, i.e. disease susceptibility/severity, risk perception, and public health measures, would have affected testing behaviour by modifying one another, in addition to their direct impact on testing behaviour. In our models, multiple variables were selected as proxies for each mechanism, and some of those variables were associated with more than one mechanism, depending on data available at the population level. While this modelling approach helped disentangle and explore testing behaviour in relation to various contextual meta-data within our conceptual framework, it limited our ability to evaluate other forms of causal relationships, for example, the indirect impact of disease susceptibility/severity and public health measures on risk perception (dotted lines in Fig. 1a). Thus, care must be taken when making causal inferences from such observational studies. Studies based on individual-level data might be better suited for exploring formally the causal relationships hypothesised here. For example, the probability of testing could be modelled at the individual level, with testing history as the outcome assumed to have various causal relationships at the individual-level (e.g. age and vaccination history) and population-level (e.g. dominant variant in circulation and public health measures in place) factors. However, it must also be noted that such studies face challenges in accounting for selection bias and factors that constantly change over the pandemic, notably the underlying epidemic trends. These complexities highlight the need to explore complementary study designs and results to further understand testing behaviour.

Secondly, there could be other factors associated with testing behaviour which were not explicitly investigated. Notably, data on LFD tests were not analysed as we considered that their association with PCR testing behaviour was explained by other variables included, specifically a confirmatory PCR testing requirement for those testing positive with LFD tests and PCR testing capacity for both infected and non-infected people. However, if compliance with the confirmatory PCR test requirement and the availability of PCR and LFD tests changed over time, the trend in LFD tests could have affected PCR testing behaviour differently during the study period. This relationship highlights the need for future research into the dynamics between LFD and PCR testing behaviour to optimise surveillance and control strategies with those two test types^4^. In addition, age- or region-specific associations with testing behaviour were not explored for some of the variables, mainly due to data unavailability at corresponding levels^45–47^. For example, we did not use age-specific parameters for the dominant variants to avoid correlations with parameters for vaccination; the vaccine rollout and Delta variant emergence occurred concurrently at the national level. Also, data for PCR testing capacity and Google Trends were not available at the regional level, although these variables could have varied between regions depending on regional epidemic situations. These data gaps suggest that future research based on our conceptual framework could be strengthened further by employing age- and region-specific data, should they become available.

Finally, the underlying epidemic trends were informed by the mean prevalence of SARS-CoV-2 swab positivity, i.e. REACT GAM fit, without accounting for its uncertainty. Thus, under- or over-estimation of the true prevalence could have affected model estimates by under- or over-estimating the number of infected and non-infected people. This was apparent during the early Delta variant circulation in people aged 20–39 years when the observed number of positive tests in NHS test data was greater than the estimated number of infected people by REACT GAM fit. In fact, the interval between REACT rounds during this period was much longer than other periods, suggesting that REACT GAM fit could have under-estimated the prevalence of SARS-CoV-2 swab positivity during this period, potentially by missing increasing epidemic trends when tests were not conducted between REACT study rounds. However, the overall impact of the underestimate is unlikely to be significant, considering that those observations comprised only 1.0% of the data points.

Our findings suggest how testing strategies could be optimised for different objectives, notably monitoring cases, controlling transmission, and clinical management. For monitoring cases, varying levels of disease susceptibility/severity and risk perception need to be considered when interpreting epidemic trends, as transmission among people expected to experience asymptomatic or mild infection could be underestimated in voluntary test data. For the transmission control objective, changes in risk perception require continuous assessments, and efforts should be made to maintain risk awareness and, thus, test participation. This will be particularly important when a new variant emerges with different virulence and transmissibility, as shown by this study during Omicron variant circulation, or when public health measures are newly introduced, with a potential impact on risk perception. For clinical management, the public needs to be well informed about the risk profile of disease susceptibility/severity in order to increase testing frequency among people who would need treatments upon infection. Finally, it must be noted that different testing objectives cannot be considered alone, as efforts made for one testing objective could have synergistic or counterproductive effects on other objectives. This need for balance highlights how testing strategies should be designed and updated constantly based on the relative importance of different testing objectives under given epidemic contexts.

In conclusion, our study highlights the usefulness of representative infection survey data, combined with voluntary testing and other meta-data, for understanding factors underpinning the complex dynamics of testing behaviour. While the underlying epidemic trends and disease susceptibility/severity were found to drive these dynamics, changes in risk perception and public health measures were also associated with testing behaviour. These illustrate that testing strategies should be designed based on the combined effect of these mechanisms on testing behaviour and the relative importance of different testing purposes for given epidemic situations.

## Methods

### Data

#### NHS testing data

The weekly number of people tested and the weekly number of people testing positive for COVID-19 by PCR tests via Pillar 1 and Pillar 2 routes were obtained from weekly statistics for NHS Test and Trace (England)^22^. These data represented COVID-19 swab testing in the UK: Pillar 1 targeted people with a clinical need, and health and care workers, and Pillar 2 targeted the wider population. The numbers were aggregated by week, region, and age group (Table 1).

**Table 1:** Use, definition, and source of variables

Variable	Category	Note	Source
**Outcome where models were fitted**			
NHS PCR test data		• Weekly numbers of positive and negative PCR test results via NHS Test and Trace Pillar 1 and Pillar 2 routes over 92 weeks between 28^th^ May 2020 and 24^th^ February 2022.	^22^
**Explanatory**			
1. **To account the underlying epidemic trends**			
REACT GAM fit		• Mean prevalence of SARS-CoV-2 swab positivity estimated from REACT test data • Used to divide the study population into infected and non-infected people. Thus, no parameter estimation was made for this variable.	^23^
2. **To assess the process of seeking and receiving a PCR test**			
age	age group 1	• ≤19 years old (Reference group)	
	age group 2	• 20–39 years old	
	age group 3	• 40–69 years old	
	age group 4	• ≥70 years old	
Alpha	< 50% of circulation	• Other periods • 10 Dec 2020 to 13 May 2021	^55^
	≥ 50% of circulation		
Delta	< 50% of circulation	• Other periods • 13 May 2021 to 09 Dec 2021	^56^
	≥ 50% of circulation		
Omicron	< 50% of circulation	• Other periods • 09 Dec 2021 to 24 Feb 2022	^56^
	≥ 50% of circulation		
vaccination	< 50% of population	• Cumulative proportion of people with the second vaccine dose • Age group-specific coefficients were used. ◦ From 12 Aug 2021 in age group 2 ◦ From 27 May 2021 in age group 3 ◦ From 15 Apr 2021 in age group 4 ◦ Age group 1 was not considered as vaccination did not reach 50%	^57^
	≥ 50% of population		
testing for event	not required	• 1 if a lateral flow device test was required for entering nightclubs and large events, 0 otherwise • 15 Dec 2021 to 27 Jan 2022	^58^
	required		
no confirmatory PCR	confirmatory PCR required	• The requirement for a confirmatory PCR on people with positive LFD test results • Required from 11 Jan 2022 • PCR testing was still required for people with symptoms until the end of the study period.	^59^
	confirmatory PCR not required		
testing capacity	Continuous	• Daily no. of PCR testing capacity averaged for each week	^57^
Google Trends	Continuous	• Google Trends index for search keyword ‘covid’ in the United Kingdom • Age group-specific coefficients were used. • The same value was assumed for all age groups.	^60^
school term	school holiday	• Applied only to age group 1 • In 2021, students returned to schools in March due to the third nationwide lockdown	^61^
	school term		
regions of England	South East (reference group)		
	East Midlands		
	East of England		
	London		
	North East		
	North West		
	South West		
	West Midlands		
	Yorkshire and the Humber		
nationwide lockdown	no lockdown	• 1st lockdown: 23 Mar 2020-23 Jun 2020 • 2nd lockdown: 5 Nov 2020-2 Dec 2020 • 3rd lockdown: 6 Jan 2021-29 Mar 2021 • As 3rd lockdown was gradually relaxed, we set that it ended when the ‘Stay at home’ order was abolished^21^, assuming the impact on testing behaviour was most substantial by this order.	^21^
	In lockdown		

#### REACT test data

REACT test data included PCR test results from random samples of the population of England between 27 April 2020 and 28 February 2022 (REACT rounds 1–18)^23^. The original data included the weekly number of PCR tests and, among them, the weekly number of positive tests in 9 regions and 8 age groups over 74 weeks. Since these original data were prepared using different age group definitions, we re-classified age groups by weighting the total number of tests and the number of positive tests by accounting for age group population sizes. After weighting, a binomial GAM was fitted to estimate the prevalence of SARS-CoV-2 swab positivity in each region and age group, REACT GAM fit, using the *gam* function of the *mgcv* package^48^ (see SI for details).

#### Covariates included

The definition and source of covariates are detailed in Table 1.

### Model

Models describing the process of people seeking and receiving a SARS-CoV-2 PCR test were fitted to NHS test data^49^. We allowed infected and non-infected people to have different likelihoods of taking a PCR test, considering that their decision to take a PCR test was likely influenced by several factors, notably the presence and severity of COVID-19 symptoms and recent contacts with those confirmed infected. Therefore, for each region, age group, and week, the study population was divided into infected and non-infected people first. The number of infected people (${N}_{R,A,W}^{1}$, i.e. producing a true positive or false negative test result if tested, orange square in the top panel of Fig. 1b) was estimated as a product of the population size and the mean prevalence of SARS-CoV-2 swab positivity from REACT GAM fit. The number of non-infected people (${N}_{R,A,W}^{2}$, i.e. producing a true negative or false positive test result if tested, blue square in the top panel of Fig. 1b) was estimated similarly. Then, the expected numbers of positive and negative PCR test results were modelled with the following structure:

1 $${n}_{R,A,W}^{p}={N}_{R,A,W}^{1}{\theta }_{R,A,W}^{1}\varphi+{{N}_{R,A,W}^{2}\theta }_{R,A,W}^{2}\left(1-\omega \right)$$

2 $${n}_{R,A,W}^{n}={{N}_{R,A,W}^{2}\theta }_{R,A,W}^{2}\omega+{N}_{R,A,W}^{1}{\theta }_{R,A,W}^{1}\left(1-\varphi \right)$$

For region, *R*, age group *A*, and week, *W*, ${n}_{R,A,W}^{p}$ and ${n}_{R,A,W}^{n}$ represent the expected number of positive and negative PCR test results, respectively, to be compared with respective NHS test results during parameter estimation based on their likelihoods (see below).

The expected number of positive test results was modelled as the sum of true and false positive test results, and in the same way for the expected number of negative test results. For the expected number of positive tests, ${n}_{R,A,W}^{p}$, the first and second terms of the right-hand side of (1) represent the expected numbers of people with true and false positive test results, respectively (red arrows in the bottom panel of Fig. 1b). The expected number of negative tests, ${n}_{R,A,W}^{n}$, was modelled in the similar manner (2) (blue arrows in the bottom panel of Fig. 1b). We assumed 95.0% and 99.9% as PCR test sensitivity ($\varphi$) and specificity ($\omega$), respectively^50^.

The probability of seeking and conducting a test was allowed to differ between infected and non-infected people, ${\theta }_{R,A,W}^{1}$ and ${\theta }_{R,A,W}^{2}$ respectively, within a logistic regression framework (Fig. 1b):

3 $${{logit}}\,({\theta _{R,A,W}^{1}})={\alpha }^{1}+\mathop{\sum}\limits_{i}{\beta }_{i}^{1}{X}_{{i}_{R,A,W}}+\mathop{\sum}\limits_{j}{\beta }_{j}^{3}{X}_{{j}_{R,A,W}}$$

4 $${{logit}}\,({\theta _{R,A,W}^{2}})={\alpha }^{2}+\mathop{\sum}\limits_{i}{\beta }_{i}^{2}{X}_{{i}_{R,A,W}}+\mathop{\sum}\limits_{j}{\beta }_{j}^{3}{X}_{{j}_{R,A,W}}$$

An intercept was estimated separately for infected and non-infected people (noted as ${\alpha }^{1}$ and$\,{\alpha }^{2}$), assuming that they had different baseline testing probabilities. The youngest age group (i.e. ≤19 years old) was chosen as the reference to present the association of age with testing behaviour in order of age and also considering its larger number of positive tests than the oldest age group (i.e. ≥70 years old). South East was chosen as the reference given its largest population among regions of England.

${X}_{R,A,W}$ represent covariates for a given region, age group, and week which were allowed to influence testing probability (see Table 1). Some variables were hypothesised to affect testing behaviour differently for infected and non-infected people (noted as ${\beta }^{1}$ and ${\beta }^{2}$, respectively). For example, while some variables may increase the testing behaviour of both infected and non-infected people by changing risk perception (blue arrows in the middle panel of Fig. 1b), they may also have an additional impact on the testing behaviour of infected people by changing the nature of the infection itself, thereby leading to greater disease susceptibility/severity (orange arrows in the middle panel of Fig. 1b).

Therefore, regression coefficients for variables likely linked to disease susceptibility/severity, i.e. *age*, *Alpha*, *Delta*, *Omicron*, and *vaccination*, were estimated separately for infected and non-infected people. Similarly, coefficients associated with *testing for event* and *no confirmatory PCR* were also estimated separately as those public health measures had different implications for taking PCR tests following positive or negative LFD test results.

In contrast, coefficients associated with variables not directly linked to disease susceptibility/severity, including *Google Trends*, *testing capacity*, *school term*, *nationwide lockdown*, and *regions of England*, were assumed the same for infected and non-infected people, i.e. prior to testing, those factors would equally affect infected and non-infected people through changes in public health measures and risk perception (noted as ${\beta }^{3}$).

In addition to *age*, the impacts of *vaccination* and *Google Trends* were also allowed to be age-specific for all age groups, and *school term* was assumed to affect age group 1 (i.e. those aged 0–19) only.

Note that age classifications in REACT test and vaccination data were slightly different from that of NHS test data. In this study, age classes were defined following age categories in NHS test data, and covariates were re-grouped into these age classes by weighting their values by the population size of each age group (See SI for details, including sensitivity analyses). A Metropolis-Hastings Markov chain Monte Carlo (MCMC) algorithm^51^ was implemented in R 4.1.2^52^ to estimate parameter based on optimising the following likelihood function, *L*:

5 $$L=\mathop{\prod}\limits_{R,A,W}{{\Pr }}\bigg({k}_{R,A,W}^{p}{{{{{\rm{;}}}}}}\;{n}_{R,A,W}^{p},\,{{{\varnothing }}}^{p}\bigg)\Pr \bigg({k}_{R,A,W}^{n}{{{{{\rm{;}}}}}}\;{n}_{R,A,W}^{n},\,{{{\varnothing }}}^{n}\bigg)$$

Here, the likelihood of observing the number of NHS positive test results, ${k}_{R,A,W}^{p}$, (or negative test results, ${k}_{R,A,W}^{n}$) was assumed to follow a negative binomial distribution with mean ${n}_{R,A,W}^{p}$ (or ${n}_{R,A,W}^{n}$) and overdispersion parameter ${\varnothing }^{p}$ (or ${\varnothing }^{n}$). Uniform prior distributions were used, except for ${\varnothing }^{p}$ and ${\varnothing }^{n}$, which were constrained to be positive. After discarding burn-in posterior estimate, 200,000 posterior samples were retained and convergence was assessed by visual inspection of MCMC trace plots and Gelman-Rubin convergence diagnostic (<1.01)^53^.

Variables were added through a manual forward-stepwise selection procedure. In the first cycle, all univariate models were considered in turn, and the model with the lowest DIC was retained, provided that the decrease in deviance was >5 units compared to the null, intercept-only, model. The same process was then repeated for the remaining variables in the next cycles until no other variables resulted in >5 DIC unit difference, leading to the best-fitting model. Posterior predictive check was performed for a model selected in each cycle by comparing the observed and simulated number of PCR-positive and -negative test results for each region, age group and week.

To assess how much variability in NHS test data was explained when variables were added incrementally, the Deviance *R*-squared was estimated for each selected model separately for the positive and negative test results and for both, accounting for overdispersed count data^54^. Additionally, ${\theta }^{1}$ and ${\theta }^{2}$ were computed accounting for all variables in the best-fitting model, to assess the trends in testing behaviour during the pandemic.

### Reporting summary

Further information on research design is available in the Nature Portfolio Reporting Summary linked to this article.

## Supplementary information

Supplementary Information

Reporting Summary

## Data Availability

This study used publicly available population-level data. Data sources are presented in Table 1.

## Code availability

Model codes are available from https://github.com/KimYounjung/COVIDTestingBehaviour.
