# Supplementary material for: Drivers of SARS-CoV-2 testing behaviour: a modelling study using nationwide testing data in England
Source: Nat Commun. 2023 Apr 14;14:2148. doi: 10.1038/s41467-023-37813-1 (PMC10103662; doi:10.1038/s41467-023-37813-1)
Supplement: Supplementary file 1 — Supplementary Information [file 41467_2023_37813_MOESM1_ESM.pdf]

Drivers of SARS-CoV-2 testing behaviour:

a modelling study using nationwide testing data in England

Younjung Kim [a], Christl A. Donnelly [b, c, d], Pierre Nouvellet [a, d,†]

a Department of Ecology, Behaviour, and Environment, School of Life Sciences, University of Sussex, Brighton, UK

b Department of Statistics, University of Oxford, Oxford, UK

c Pandemic Sciences Institute, University of Oxford, Oxford, UK

d MRC Centre for Global Infectious Disease Analysis and Abdul Latif Jameel Institute for Disease and Emergency Analytics, Imperial College London, London, UK

† Corresponding author: pierre.nouvellet@sussex.ac.uk

Contents

Supplementary Methods 2

Weighting of REACT test data 2

Estimation of prevalence of SARS-CoV-2 swab positivity based on REACT test data 2

Weighting of vaccination data 2

Supplementary Tables and Figures 3

Table S1 Odds ratio for taking a SARS-CoV-2 PCR test from the best-fitting model. Variables specific to infected or non-infected people 3

Table S2 Odds ratio for taking a SARS-CoV-2 PCR test from the best-fitting model. Variables for both infected and non-infected people 4

Table S3 Age classification of NHS test data and weighted REACT test data 4

Figure S1 Prevalence of SARS-CoV-2 swab positivity estimated from REACT test data 5

Figure S2 Predictive posterior check of models selected during the forward stepwise selection procedure, by age group and over time 6

Figure S3 Predictive posterior check of the best-fitting model by England region and over time.... 7-9

Figure S4 Predictive posterior check of models selected during the forward stepwise selection procedure, by England region and over time 10-12

Figure S5 Probability of taking a SARS-CoV-2 PCR test by age group and over time 13

Figure S6 Sensitivity analyses of odds ratio for taking a SARS-CoV-2 PCR test 14

Supplementary Methods

Weighting of REACT test data

REACT test data included PCR test results from random samples of the population of England between 27 April 2020 and 28 February 2022 (REACT rounds 1-18). The original data included the total number of PCR tests and, among them, the number of positive tests stratified by 9 regions and 8 age groups. Since these original data were prepared using different age group definitions, we re-classified age groups by weighting the total number of tests and the number of positive tests by accounting for age group population sizes (Table S3). For example, the original data had an age group for people aged 18-24 years. Since the upper limit of this age group was not the same as that of age group 1 of NHS test data, we assumed that a fraction of PCR tests and a fraction of positive tests were from people aged 18-19 years, and the remaining fraction from people aged 20-24 years, following the relative population size of those two age groups. This weighting method was applied to other age groups in the same way. (see Fig. S5 for sensitivity analysis results from the best-fitting model with and without weighting).

Estimation of prevalence of SARS-CoV-2 swab positivity based on REACT test data

A binomial generalised additive model (GAM) was fitted to REACT test data to estimate the prevalence of SARS-CoV-2 swab positivity in each region and age group. The GAM fitted one smooth and one linear term for each region-age group combination, assuming that epidemic trends varied between regions and age groups. Based on GAM estimates, the mean prevalence of SARS-CoV-2 swab positivity was predicted over the study period and provided as REACT GAM fit.

Weighting of vaccination data

Like REACT test data, vaccination data used different age group definitions, but only for one age group. Vaccination data in this age group were split into two, people aged 18-19 and 20-24, by weighting the number of people with second vaccination completion by the relative population size of those two age groups (Table S3). (see Fig. S6 for sensitivity analysis results from the best-fitting model with and without weighting).

Supplementary Tables and Figures

Table S1 Odds ratio for taking a SARS-CoV-2 PCR test from the best-fitting model. Variables specific to infected or non-infected people [a]

Variable	Odds ratio			
	Infected people		Non-infected people	
	Median	95% CrI	Median	95% CrI
Intercept	0.04	0.04-0.04	0.00	0.00-0.00
age				
age group 2 (20 to 39 years old)	3.49	3.12-3.91	2.85	2.65-3.06
age group 3 (40 to 69 years old)	3.01	2.70-3.40	2.81	2.61-3.01
age group 4 (≥70 years old)	6.49	5.73-7.34	3.72	3.48-3.98
Alpha [b]	0.78	0.71-0.85	0.66	0.64-0.69
Delta [b]	1.63	1.46-1.82	1.05	1.01-1.09
Omicron [b]	0.96	0.74-1.26	0.65	0.59-0.70
vaccination [c]				
age group 2 (20 to 39 years old)	0.57	0.48-0.66	0.83	0.79-0.86
age group 3 (40 to 69 years old)	0.71	0.61-0.83	0.92	0.88-0.96
age group 4 (≥70 years old)	0.23	0.20-0.27	0.86	0.83-0.90
testing for event	1.30	1.09-1.54	1.06	1.00-1.13
no confirmatory PCR	0.28	0.22-0.34	0.81	0.75-0.87

a Infected (or non-infected) people aged ≤19 years (age group 1) in South East, with other variables kept minimum or not in place, represented the reference group for parameters estimated for infected (or non-infected) people.

b Odds ratio linked to ≥50% of circulating SARS-CoV-2 being a given variant

c Odds ratio linked to ≥50% of the population receiving a second dose of vaccination

Table S2 Odds ratio for taking a SARS-CoV-2 PCR test from the best-fitting model. Variables for both infected and non-infected people [a]

Variable	Odds ratio	
	Median	95% CrI
Google Trends		
age group 1 (≤19 years old)	2.64	2.32-2.97
age group 2 (20 to 39 years old)	3.76	3.30-4.28
age group 3 (40 to 69 years old)	3.26	2.88-3.72
age group 4 (≥70 years old)	1.41	1.25-1.59
testing capacity	4.47	4.18-4.79
nationwide lockdown	0.83	0.81-0.86
school term	1.57	1.52-1.62
regions of England		
East Midlands	0.91	0.88-0.94
East of England	0.97	0.94-1.00
London	0.99	0.96-1.02
North East	0.90	0.87-0.93
North West	0.99	0.96-1.03
South West	0.97	0.94-1.01
West Midlands	0.90	0.87-0.93
Yorkshire and the Humber	0.88	0.85-0.91

a Infected (or non-infected) people aged ≤19 years (age group 1) in South East, with other variables kept minimum or not in place, represented the reference group for parameters estimated for infected (or non-infected) people.

Table S3 Age classification of NHS test data and weighted REACT test data

	NHS test data	REACT study data (weighted)	Vaccination data (weighted)
Age group 1	≤19	5-17, (18-19)	12-17, (18-19)
Age group 2	20-39	(20-24), 25-34, (35-39)	(20-24), 25-39
Age group 3	40-69	(40-44), 45-64, (65-69)	40-69
Age group 4	≥70	(≥70)	≥70

Original REACT study data age groups: 5-13, 13-17, 18-24, 25-34, 35-44, 45-54, 55-64, 65+

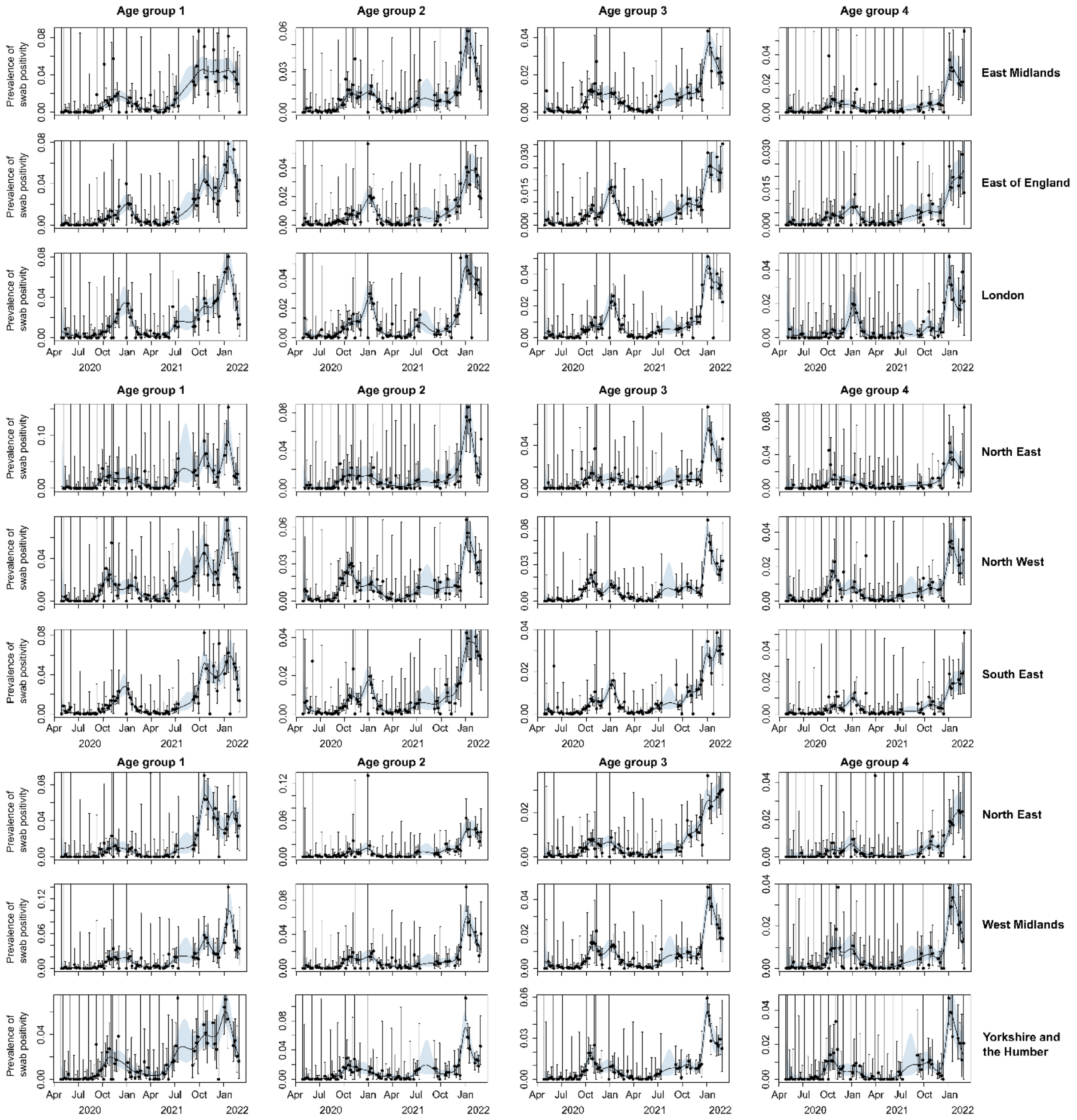

Figure S1 Prevalence of SARS-CoV-2 swab positivity estimated from REACT test data. Points and vertical lines represent the mean proportion of PCR-positive test results from REACT test data and its 95% confidence intervals, respectively. Curved lines represent the mean prevalence predicted by fitting a binomial generalised additive model (GAM) to REACT test data (see SI for details), with blue shades representing its 95% confidence intervals. The predictions were made over the study period for each region (rows) and age group (columns).

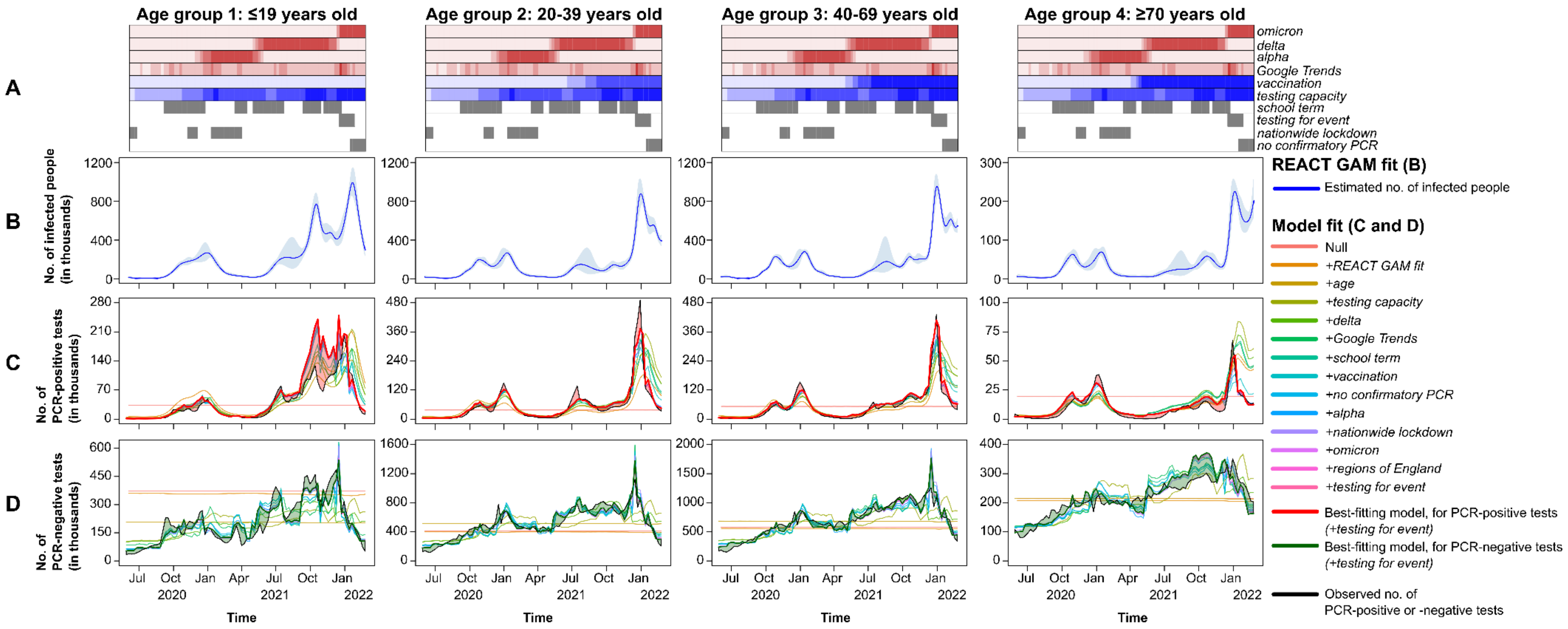

Figure S2 Predictive posterior check of models selected during the forward stepwise selection procedure, by age group and over time. Row **A** shows the temporal trend of variables included in the best-fitting model, and Row **B** shows the temporal trend of the number of infected (mean, 95% confidence intervals) estimated by REACT generalised additive model (GAM) fit. Row **C** shows the predicted (lines: median, shades: 95% percentile intervals) and observed numbers of Pillar 1 and Pillar 2 PCR-positive test results. Row **D** shows the predicted (lines: median, shades: 95% percentile intervals) and observed (black lines) numbers of Pillar 1 and Pillar 2 PCR-negative test results. For predicted values, different colours represented different models selected during a manual forward stepwise selection procedure.

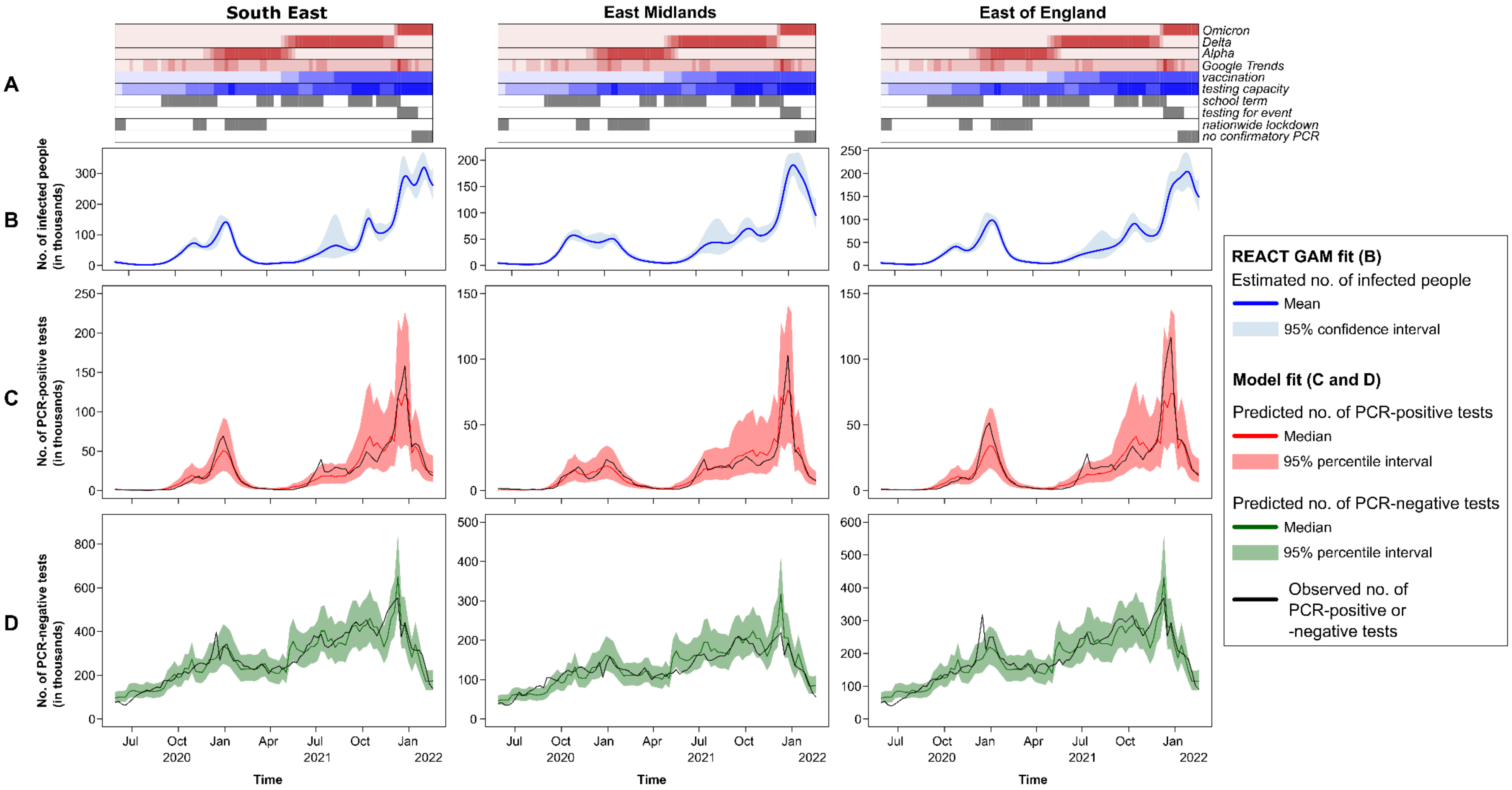

Figure S3 Predictive posterior check of the best-fitting model by region (South East, East Midlands, and East of England) and over time. For each panel, Row **A** shows the temporal trend of variables included in the best-fitting model, and Row **B** shows the temporal trend of the number of infected (mean, 95% confidence intervals) estimated by REACT generalised additive model (GAM) fit. Row **C** shows the predicted (red lines: median, reddish shades: 95% percentile intervals) and observed (black lines) numbers of Pillar 1 and Pillar 2 PCR-positive test results. Row **D** shows the predicted (green lines: median, greenish shades: 95% percentile intervals) and observed (black lines) numbers of Pillar 1 and Pillar 2 PCR-negative test results.

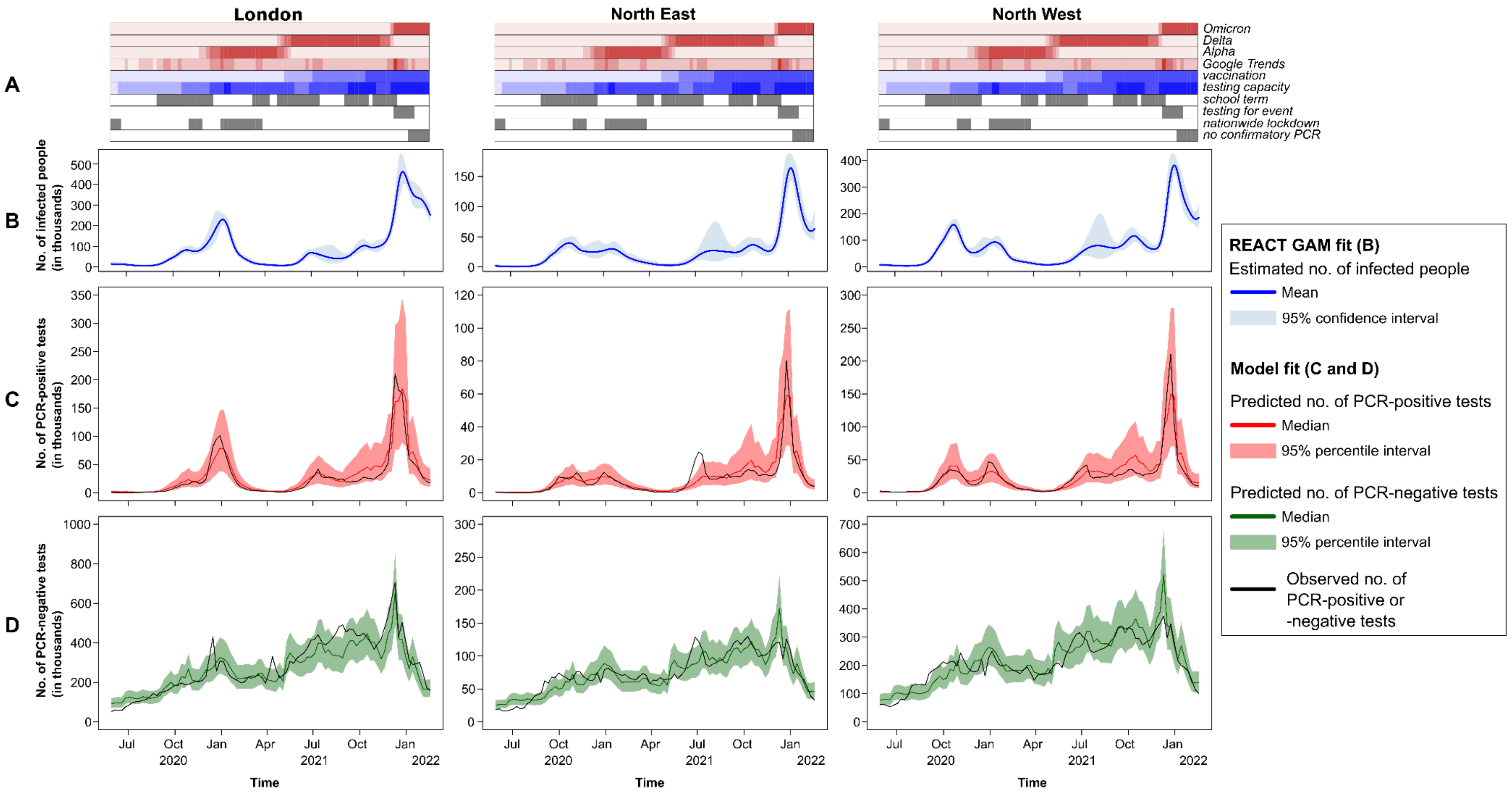

Figure S3 Predictive posterior check of the best-fitting model by region (London, North East, and North West) and over time. For each panel, Row **A** shows the temporal trend of variables included in the best-fitting model, and Row **B** shows the temporal trend of the number of infected (mean, 95% confidence intervals) estimated by REACT generalised additive model (GAM) fit. Row **C** shows the predicted (red lines: median, reddish shades: 95% percentile intervals) and observed (black lines) numbers of Pillar 1 and Pillar 2 PCR-positive test results. Row **D** shows the predicted (green lines: median, greenish shades: 95% percentile intervals) and observed (black lines) numbers of Pillar 1 and Pillar 2 PCR-negative test results.

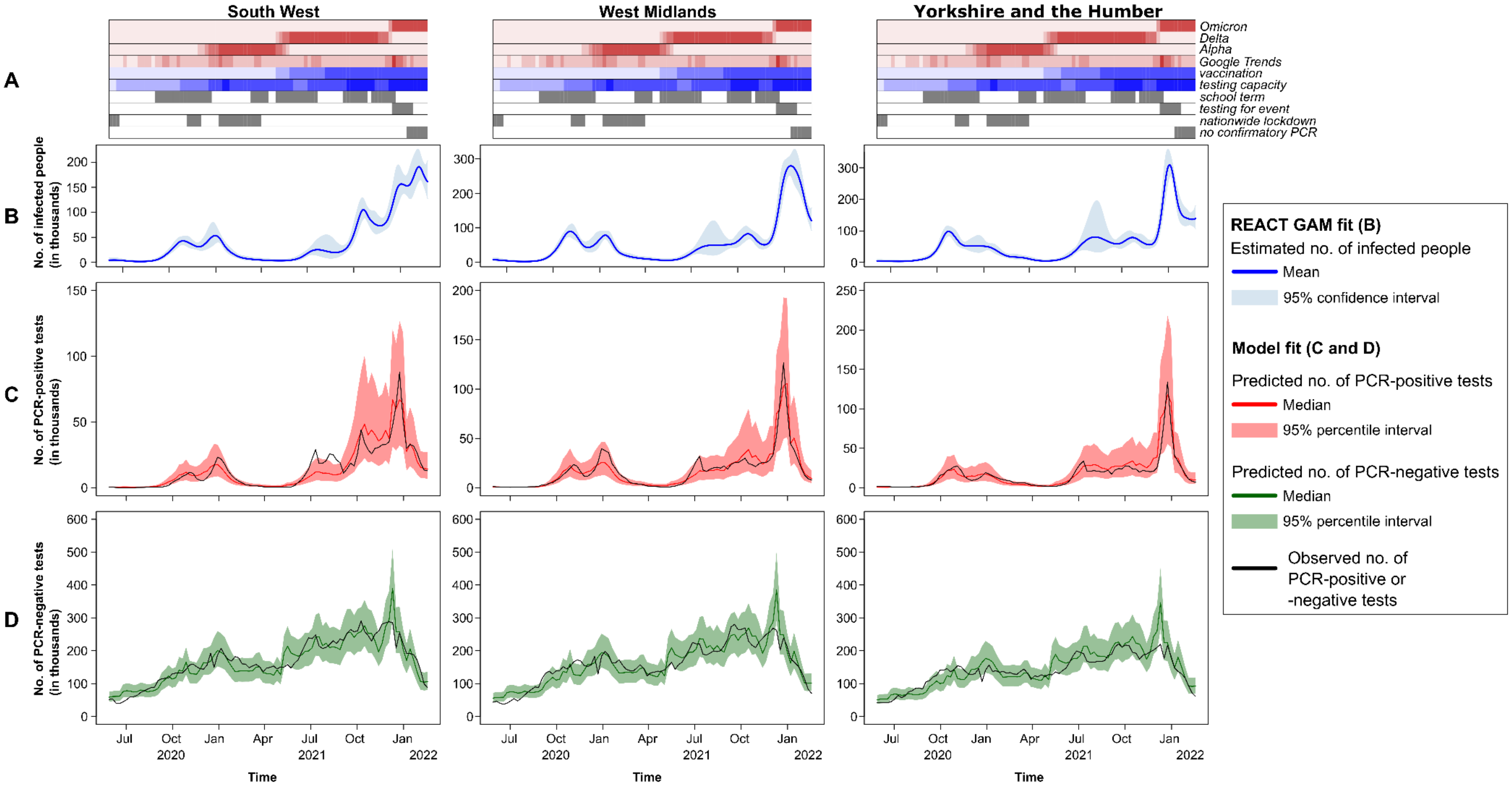

Figure S3 Predictive posterior check of the best-fitting model by region (South West, West Midlands, Yorkshire and the Humber) and over time. For each panel, Row **A** shows the temporal trend of variables included in the best-fitting model, and Row **B** shows the temporal trend of the number of infected (mean, 95% confidence intervals) estimated by REACT generalised additive model (GAM) fit. Row **C** shows the predicted (red lines: median, reddish shades: 95% percentile intervals) and observed (black lines) numbers of Pillar 1 and Pillar 2 PCR-positive test results. Row **D** shows the predicted (green lines: median, greenish shades: 95% percentile intervals) and observed (black lines) numbers of Pillar 1 and Pillar 2 PCR-negative test results.

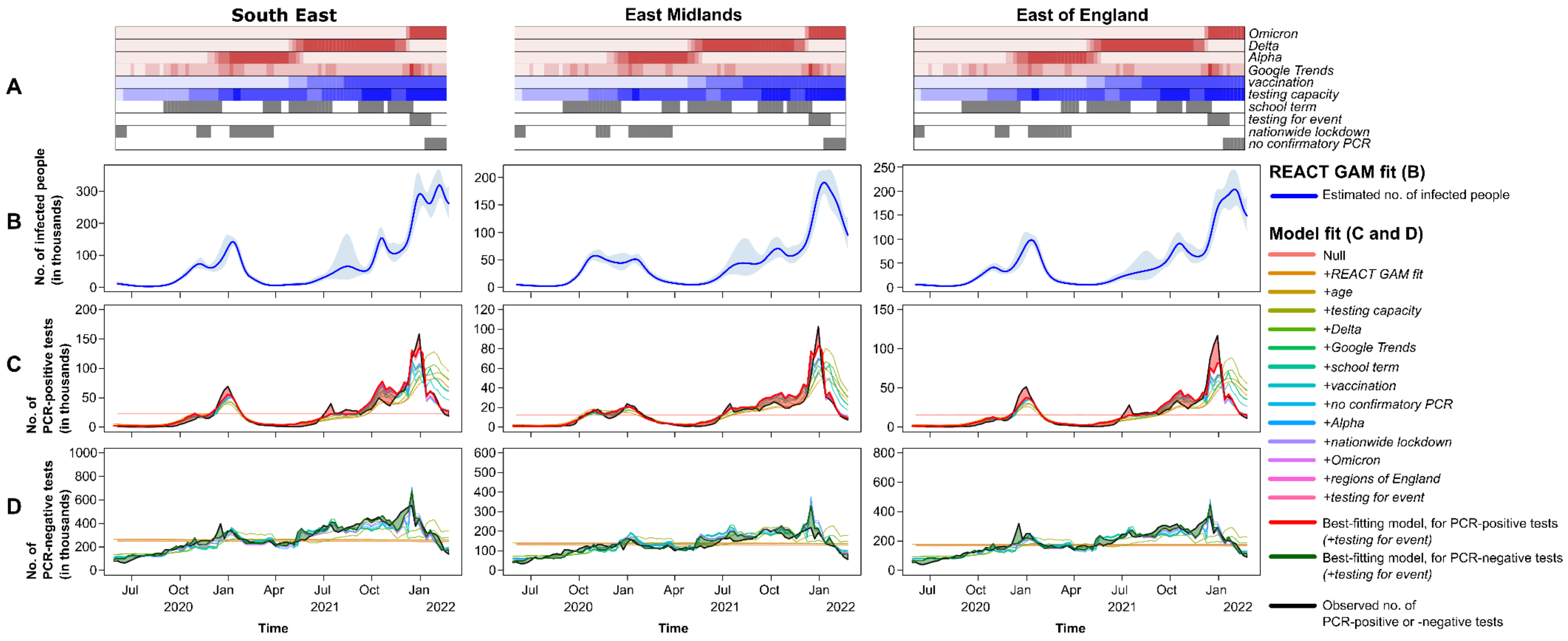

Figure S4 Predictive posterior check of models selected during the forward stepwise selection procedure, by region (South East, East Midlands, and East of England) and over time. Row **A** shows the temporal trend of variables included in the best-fitting model, and Row **B** shows the temporal trend of the number of infected (mean, 95% confidence intervals) estimated by REACT generalised additive model (GAM) fit. Row **C** shows the predicted (lines: median, shades: 95% percentile intervals) and observed numbers of Pillar 1 and Pillar 2 PCR-positive test results. Row **D** shows the predicted (lines: median, shades: 95% percentile intervals) and observed (black lines) numbers of Pillar 1 and Pillar 2 PCR-negative test results. For predicted values, different colours represented different models selected during a manual forward stepwise selection procedure.

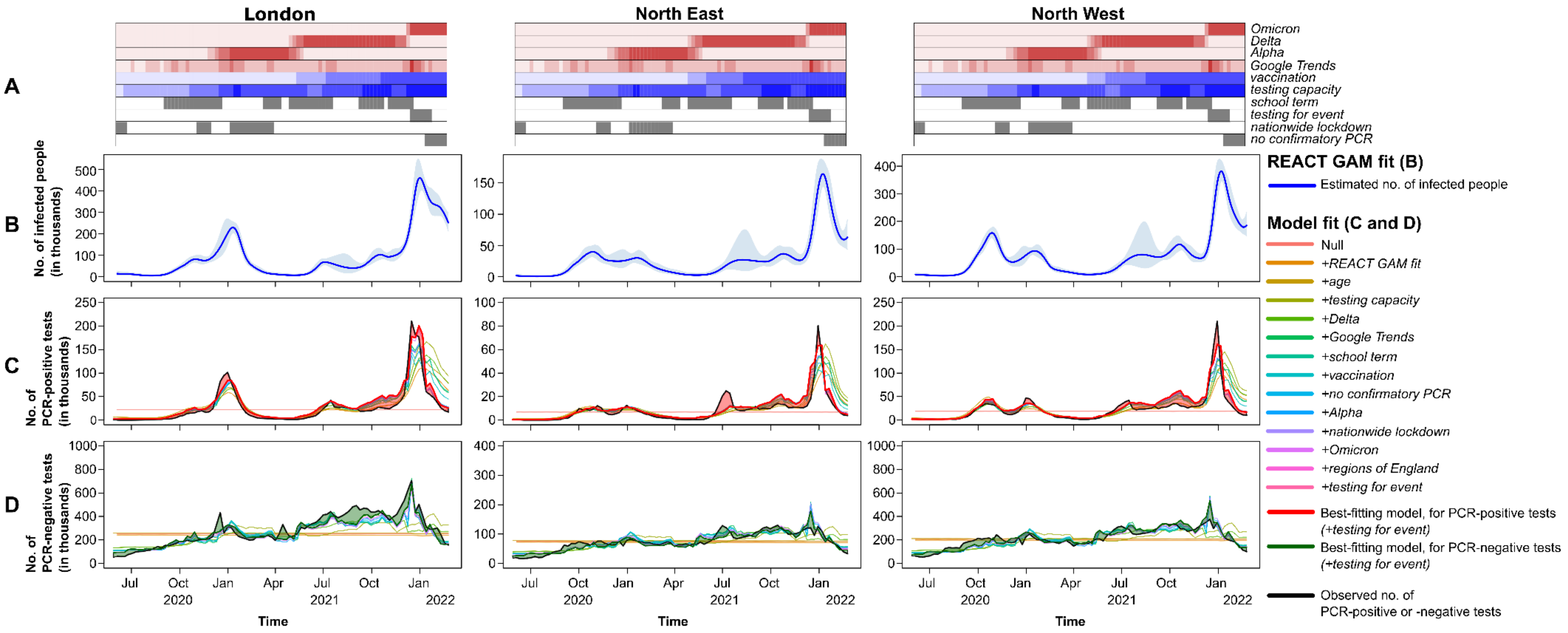

Figure S4 Predictive posterior check of models selected during the forward stepwise selection procedure, by region (London, North East, and North West) and over time. Row **A** shows the temporal trend of variables included in the best-fitting model, and Row **B** shows the temporal trend of the number of infected (mean, 95% confidence intervals) estimated by REACT generalised additive model (GAM) fit. Row **C** shows the predicted (lines: median, shades: 95% percentile intervals) and observed numbers of Pillar 1 and Pillar 2 PCR-positive test results. Row **D** shows the predicted (lines: median, shades: 95% percentile intervals) and observed (black lines) numbers of Pillar 1 and Pillar 2 PCR-negative test results. For predicted values, different colours represented different models selected during a manual forward stepwise selection procedure.

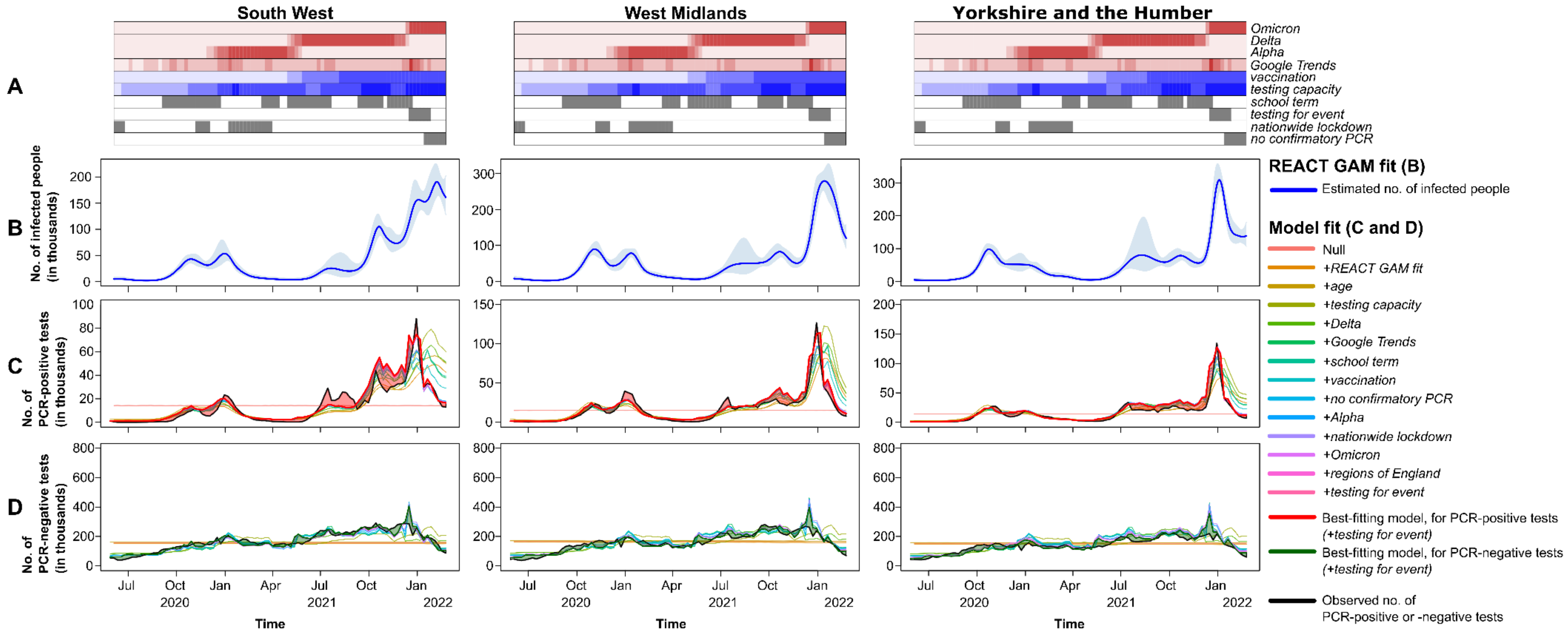

Figure S4 Predictive posterior check of models selected during the forward stepwise selection procedure, by region (South West, West Midlands, and Yorkshire and the Humber) and over time. Row **A** shows the temporal trend of variables included in the best-fitting model, and Row **B** shows the temporal trend of the number of infected (mean, 95% confidence intervals) estimated by REACT generalised additive model (GAM) fit. Row **C** shows the predicted (lines: median, shades: 95% percentile intervals) and observed numbers of Pillar 1 and Pillar 2 PCR-positive test results. Row **D** shows the predicted (lines: median, shades: 95% percentile intervals) and observed (black lines) numbers of Pillar 1 and Pillar 2 PCR-negative test results. For predicted values, different colours represented different models selected during a manual forward stepwise selection procedure.

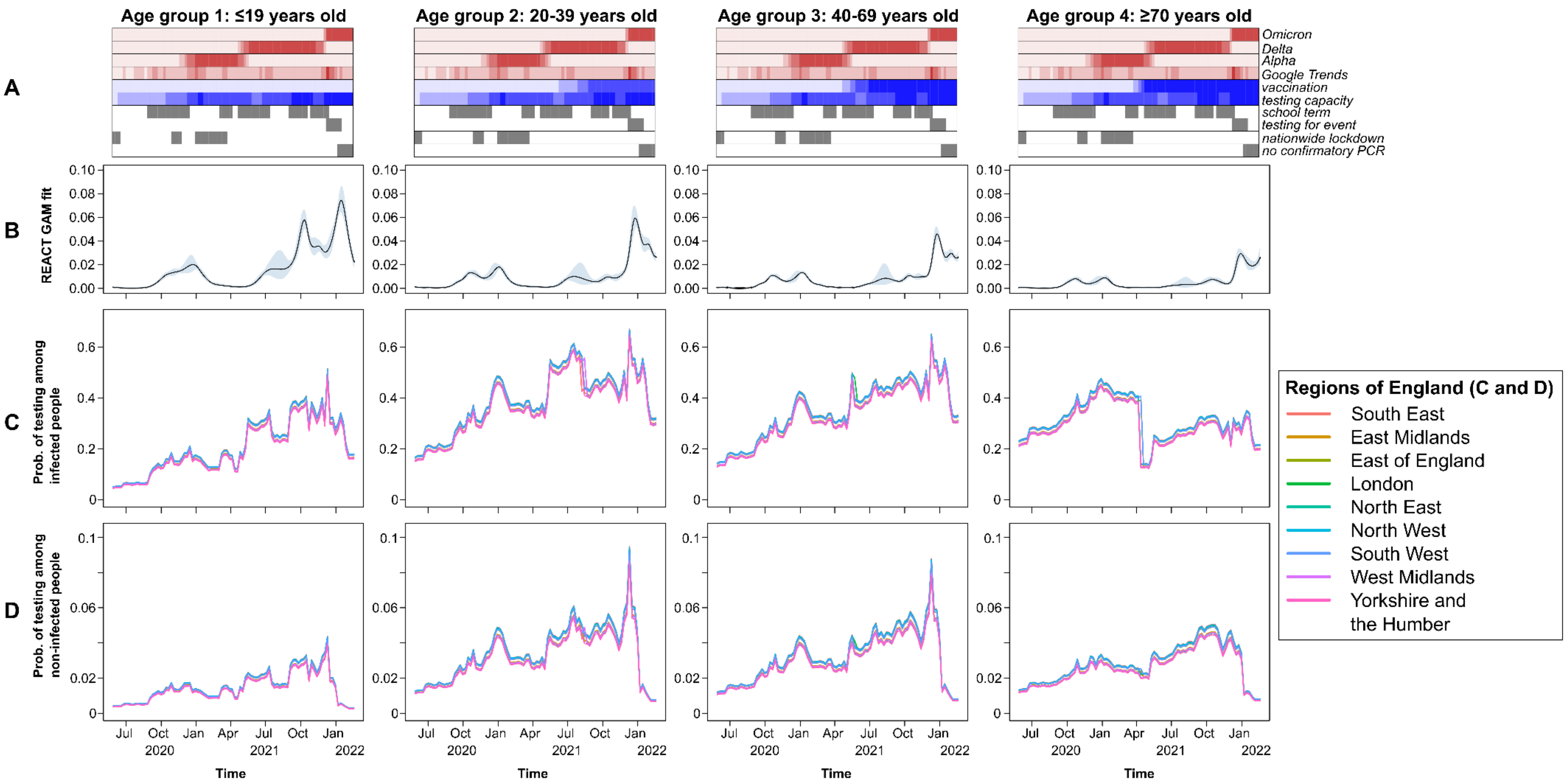

Figure S5 Probability of taking a SARS-CoV-2 PCR test by age group and over time. Row **A** shows the temporal trend of variables included in the best-fitting model, and Row **B** shows the temporal trend of REACT GAM fit (lines: mean, shades: 95% confidence intervals). Rows **C** and **D** show the probability of testing among the infected and non-infected, respectively, by age group and over time, estimated by the best-fitting model. Different line colours represent median values for different regions.

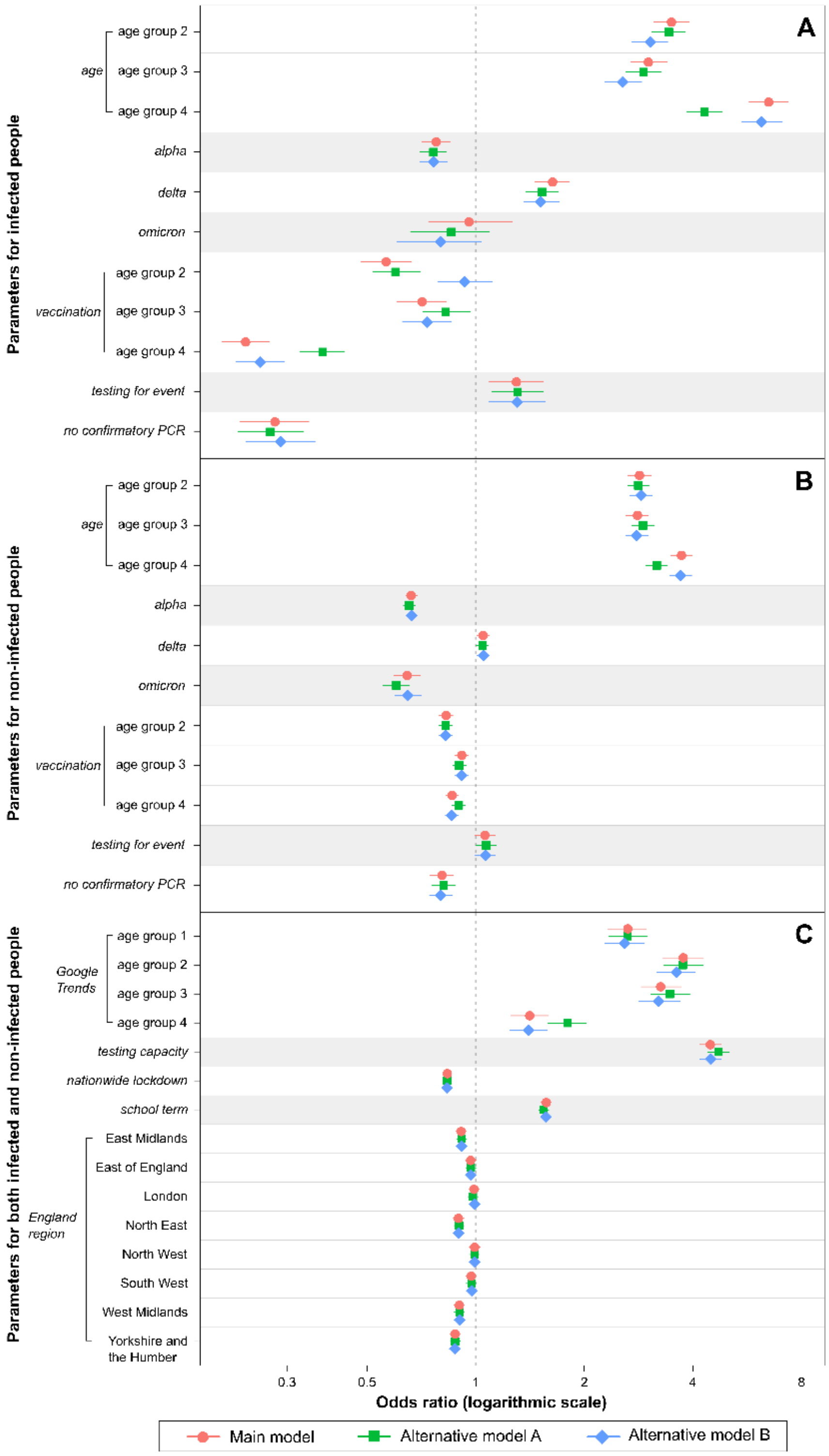

Figure S6 Sensitivity analyses of odds ratio for taking a SARS-CoV-2 PCR test. The x-axis shows odds ratios on a logarithmic scale, and the y-axis shows variables. Points and horizontal lines correspond to median and 95% credible intervals, respectively, estimated for the infected (**A**), non-infected (**B**), or without differentiation (**C**). Different colours represent the odds ratio estimated by different models. The main model corresponds to the best-fitting model presented in the main text. While people aged ≥70 years represented age group 4 of the main model, the cut-off was decreased to ≥60 years in alternative model A. Both the main model and alternative model A accounted for age group differences from REACT study and vaccination data by weighting the data based on population sizes. Alternative model B had the same age

classification as the main model, but weighting was not made. The infected (or non-infected) in age group 1 in South East, with other variables kept minimum or not in place, represented the reference group for parameters estimated for the infectious (or non-infected). Parameters estimated for both the infectious and non-infectious had the same reference group with no differentiation in infection status. For testing capacity and Google Trends, odds ratios comparing the maximum and minimum values are shown. The models in this study were fitted to weekly NHS test data for England, which ranged from 392,873 (18 to 24 Jun 2020) to 3,835,758 (16 to 22 Dec 2021) tests over 92 weeks. See UK Health Security Agency[22] for NHS test data.
