# Supplementary material for: Drivers of SARS-CoV-2 testing behaviour: a modelling study using nationwide testing data in England
Source: Nat Commun. 2023 Apr 14;14:2148. doi: 10.1038/s41467-023-37813-1 (PMC10103662; doi:10.1038/s41467-023-37813-1)
Supplement: Supplementary file 2 — Reporting Summary [file 41467_2023_37813_MOESM2_ESM.pdf]

nature portfolio

Corresponding author(s): Professor Pierre Nouvellet

Last updated by author(s): 27/3/23

Reporting Summary

Nature Portfolio wishes to improve the reproducibility of the work that we publish. This form provides structure for consistency and transparency in reporting. For further information on Nature Portfolio policies, see our Editorial Policies and the Editorial Policy Checklist.

Statistics

For all statistical analyses, confirm that the following items are present in the figure legend, table legend, main text, or Methods section.

n/a	Confirmed	
☐	☒	The exact sample size (*n*) for each experimental group/condition, given as a discrete number and unit of measurement
☐	☒	A statement on whether measurements were taken from distinct samples or whether the same sample was measured repeatedly
☐	☒	The statistical test(s) used AND whether they are one- or two-sided<br>*Only common tests should be described solely by name; describe more complex techniques in the Methods section.*
☐	☒	A description of all covariates tested
☐	☒	A description of any assumptions or corrections, such as tests of normality and adjustment for multiple comparisons
☐	☒	A full description of the statistical parameters including central tendency (e.g. means) or other basic estimates (e.g. regression coefficient) AND variation (e.g. standard deviation) or associated estimates of uncertainty (e.g. confidence intervals)
☒	☐	For null hypothesis testing, the test statistic (e.g. *F*, *t*, *r*) with confidence intervals, effect sizes, degrees of freedom and *P* value noted<br>*Give P values as exact values whenever suitable.*
☐	☒	For Bayesian analysis, information on the choice of priors and Markov chain Monte Carlo settings
☐	☒	For hierarchical and complex designs, identification of the appropriate level for tests and full reporting of outcomes
☐	☒	Estimates of effect sizes (e.g. Cohen's *d*, Pearson's *r*), indicating how they were calculated

Our web collection on statistics for biologists contains articles on many of the points above.

Software and code

Policy information about availability of computer code

Data collection	No software code was used to collect data.
Data analysis	Models describing COVID-19 PCR testing behaviour were coded and fitted to data in R software (version 4.1.2). For the analysis of generalised additive models, the gam function of the mgcv package was used in R software (version 4.1.2). The custom code is provided in Github (https://github.com/KimYounjung/COVIDTestingBehaviour).

For manuscripts utilizing custom algorithms or software that are central to the research but not yet described in published literature, software must be made available to editors and reviewers. We strongly encourage code deposition in a community repository (e.g. GitHub). See the Nature Portfolio guidelines for submitting code & software for further information.

Data

Policy information about availability of data

All manuscripts must include a data availability statement. This statement should provide the following information, where applicable:

- Accession codes, unique identifiers, or web links for publicly available datasets
- A description of any restrictions on data availability
- For clinical datasets or third party data, please ensure that the statement adheres to our policy

The present study used publicly available population-level data. Data sources are described in Table 1.

Human research participants

Policy information about studies involving human research participants and Sex and Gender in Research.

Reporting on sex and gender	The present study did not collect and analyse sex and gender information.
Population characteristics	The present study used publicly available population-level data for England disaggregated by region, age group, week.
Recruitment	Not applicable. The present study used publicly available population-level data for England.
Ethics oversight	The ethics approval was not required as the present study used publicly available population-level data.

Note that full information on the approval of the study protocol must also be provided in the manuscript.

Field-specific reporting

Please select the one below that is the best fit for your research. If you are not sure, read the appropriate sections before making your selection.

☐ Life sciences ☒ Behavioural & social sciences ☐ Ecological, evolutionary & environmental sciences

For a reference copy of the document with all sections, see nature.com/documents/nr-reporting-summary-flat.pdf

Behavioural & social sciences study design

All studies must disclose on these points even when the disclosure is negative.

Study description	The present study analysed COVID-19 testing behaviour by fitting mathematical models describing the process of people seeking and receiving a COVID-19 PCR test to nationwide surveillance testing data.
Research sample	The present study used publicly available population-level data for England disaggregated by region, age group, week.
Sampling strategy	Not applicable. The present study used publicly available population-level data for England.
Data collection	The present study used publicly available population-level data for England. Data sources are described in Table 1.
Timing	The present study used publicly available population-level data for England representing between 28/05/2020 and 24/02/2022.
Data exclusions	Not applicable. The present study used publicly available population-level data for England.
Non-participation	Not applicable. The present study used publicly available population-level data for England.
Randomization	Not applicable. The present study used publicly available population-level data for England.

Reporting for specific materials, systems and methods

We require information from authors about some types of materials, experimental systems and methods used in many studies. Here, indicate whether each material, system or method listed is relevant to your study. If you are not sure if a list item applies to your research, read the appropriate section before selecting a response.

Materials & experimental systems

n/a	Involved in the study	
☒	☐	Antibodies
☒	☐	Eukaryotic cell lines
☒	☐	Palaeontology and archaeology
☒	☐	Animals and other organisms
☒	☐	Clinical data
☒	☐	Dual use research of concern

Methods

n/a	Involved in the study	
☒	☐	ChIP-seq
☒	☐	Flow cytometry
☒	☐	MRI-based neuroimaging
